# Clinical and laboratory data of a large series of patients with congenital generalized lipodystrophy

**DOI:** 10.1186/s13098-016-0140-x

**Published:** 2016-03-15

**Authors:** Josivan G. Lima, Lucia Helena C. Nobrega, Natalia Nobrega de Lima, Maria Goretti do Nascimento Santos, Maria F. P. Baracho, Selma Maria Bezerra Jeronimo

**Affiliations:** Departamento de Medicina Clínica, Hospital Universitário Onofre Lopes (HUOL)/UFRN, Av. Nilo Peçanha, 620 - Petrópolis, Natal, RN 59012-300 Brazil; Health Graduate Program, Natal, Brazil; Departamento de Análises Clínicas e Toxicológicas, UFRN, Natal, Brazil; Instituto de Medicina Tropical do Rio Grande do Norte, Natal, Brazil; Departamento de Bioquímica, Centro de Biociências, Universidade Federal do Rio Grande do Norte, Natal, RN Brazil; Institute of Science and Technology of Tropical Diseases, INCT-DT, Salvador, Brazil

**Keywords:** Lipodystrophy, Diabetes, Insulin resistance, Berardinelli-Seip

## Abstract

**Background:**

Berardinelli-Seip congenital lipodystrophy (BSCL) was initially described by Berardinelli in Brazil in 1954 and 5 years later by Seip in Norway. It is an autosomal recessive disease that leads to a generalized deficit of body fat, evolving with diabetes and hypertriglyceridemia. The aim of this study was to describe the clinical and laboratory characteristics of a large series of patients with BSCL.

**Methods:**

This is a cross-sectional study of patients with BSCL. A total of 54 cases of BSCL were diagnosed, treated and followed for the past 17 years. We report clinical and laboratorial data of 44 of those patients.

**Results:**

There was a predominance of female patients (27 patients), and the mean age was 21.3 ± 13.7 years old. The majority of patients (30/44; 68.2 %) were diabetic, and almost half of them (14/30 patients, 46.7 %) were on insulin. The mean body mass index was 19.6 ± 3.3 and the mean body fat measured by dual-energy X-ray absorptiometry (DEXA) was 5.4 ± 0.8 %. Acanthosis nigricans, acromegaloid facies, atrophic cheeks, prognathism, phlebomegaly, and muscle hypertrophy were the most common clinical features. Only two patients had triglyceridemia lower than 150 mg/dl without the use of lipid-lowering drugs. Hyperinsulinemia was present in the majority of patients, and leptin values were very low in all patients.

**Conclusions:**

We report one of the largest series of patients with BSCL treated at a single medical center. Earlier identification of the mutations and a better understanding of the pathophysiology can aid to better treatment and decrease complications, potentially improving life quality and expectancy.

## Background

Diabetes, especially Type 2, has become a world health problem, usually resulting from excessive weight and increased visceral fat. However, other less common forms of diabetes occur, some because of specific genetic alterations. Congenital generalized lipodystrophy (CGL) was first described in Brazil in 1954 by Waldemar Berardinelli in two children [1], and 5 years later Martin Seip in Norway also described three other similar cases [2]. Berardinelli-Seip congenital lipodystrophy (BSCL) is clinically characterized by hepatosplenomegaly, fatty liver, altered carbohydrate metabolism, severe insulin resistance, hyperinsulinemia, acromegaloid habitus, and dyslipidemia.

BSCL clinical characteristics and outcome are relatively well known, and new mutations recently described have allowed a better understanding of the disease’s pathophysiology. Currently, the disease is classified into four types according to clinical characteristics and the type of mutations. Genes involved in the pathophysiology of BSCL are responsible for adipogenesis and lipogenesis, including synthesis of triacylglycerol [3], fusion of lipid droplets [4], development and maturation of adipocytes [5].

The BSCL is an autosomal recessive syndrome with around 500 cases reported in the world [6, 7]. In Brazil, in the State of Rio Grande do Norte, we have diagnosed, treated, and followed 54 cases of BSCL in the past 17 years. The aim of this study was to describe the clinical and laboratory characteristics of a large series of patients with BSCL, correlating the findings with the pathophysiology of the disease.

## Methods

### Patients

We report clinical, laboratory, and genetic features of 44 patients with CGL who are followed at the endocrine clinic of Hospital Universitário Onofre Lopes, Natal-RN, Brazil. As the disease has an autosomal recessive inheritance, our large number of patients is due to consanguinity, common in the region. Furthermore, with the increase in the number of diagnosed patients, it was easier to detect similar cases in the population.

### Variables studied

Data were obtained from history and physical examination during a regular visit to the outpatient endocrine clinic at Hospital Universitário Onofre Lopes or collected retrospectively from hospital records. Arterial hypertension was considered if the patient was on antihypertensive drug treatment or if repeated blood pressure measurements were higher than 140 × 90 mmHg. Hypertriglyceridemia was diagnosed if serum triglycerides were higher than 150 mg/dl and/or patient was on lipid-lowering drugs (fibrates). Criteria of American Diabetes Association (ADA) were considered for the diagnosis of diabetes [8].

### Laboratory and image investigations

Glycemia, glycated hemoglobin (HbA1c), total cholesterol, HDL cholesterol, LDL cholesterol (calculated by Friedewald formula if triglyceride level was lower than 400 mg/dl), non-HDL cholesterol (calculated by total cholesterol minus HDL cholesterol), triglyceridemia, urea, creatinine, insulin, and leptin were measured in serum after an overnight fasting. HOMA_IR_ (homeostasis model assessment) was calculated (fasting glycemia (mmol/L) × fasting insulin (μU/ml))÷22.5) [9] and a value higher than 2.7 was considered as indicative of insulin resistance [10]. To evaluate liver damage by steatosis, aspartate aminotransferase (AST), alanine aminotransferase (ALT) and, gamma-glutamyl transpeptidase (GGT) were measured. Genetic analysis was performed as described elsewhere [11]. Body fat percentage measured by DEXA scan (Lunar ^®^ DPX) was available from 20 patients. Liver biopsy and ultrasound were not performed.

### Statistical methods

Parametric data are expressed as mean ± standard deviation and non-parametric data are expressed as median (minimum–maximum). Proportions are presented as n (%). T test was used to compare mean of parametric data, and Mann–Whitney test was used for non-parametric data. The Shapiro–Wilk test was used to analyze normality data distribution. To evaluate the correlation between two variables, Pearson (parametric variables) and Spearman (nonparametric variables) correlation coefficients were used. A p value <0.05 was considered statistically significant. We used SPSS, version 22, to analyze the data.

## Results

### Subjects and family history

Clinical characteristics of patients are shown in Table 1. There was a predominance of female patients (27 patients, 61.4 %) and the mean age was 21.3 ± 13.7 years old. Consanguinity between parents was reported by 72.4 % of patients with available data. There were no differences in anthropometric and laboratory findings of patients with and without parents’ consanguinity (p > 0.05). Twenty-seven patients (61.4 %) had, at least, one relative affected by the syndrome. Regarding the lipid and glucose control, having a positive family history of the syndrome did not confer a worse outcome of the disease. The vast majority of patients (30/44; 68.2 %) were diabetic, and almost half of them (14 patients, 46.7 %) were on insulin. The mean age of diabetes onset was 15.8 ± 7.1 years old. Diabetics were older than non-diabetics (27.3 ± 11.0 vs. 8.5 ± 9.6 year-old, p < 0.001). The mean duration of diabetes was 11.8 ± 7.3 years. Arterial hypertension, defined as the use of antihypertensive drugs, was present in 12 patients (27.2 %).

**Table 1:** Clinical characteristics of patients

	Mean ± SD	n (%)
Age (years)	21.3 ± 13.7	
Gender	–	
Female	–	27 (61.4)
Male	–	17 (38.6)
Diabetes	–	30 (68.2)
Arterial hypertension	–	12 (27.3)
Hypertriglyceridemia	–	42 (95.5)
Body mass index (Kg/m^2^)	19.6 ± 3.3	–
Body fat (%)	5.4 ± 0.8	–
Waist (cm)	76.4 ± 8.8	–
Waist/hip ratio	0.93 ± 0.07	–
Heart rate (bpm)	85.3 ± 10.7	–
Systolic blood pressure (mmHg)^b^	122.0 ± 16.2	–
Diastolic blood pressure (mmHg)^b^	75.4 ± 9.7	–
Acanthosis nigricans	–	23 (79.3)^a^
Acromegaloid facies	–	42 (95.5)
Atrophic cheeks	–	43 (97.7)
Prognathism	–	40 (90.9)
Muscle hypertrophy	–	35 (79.5)
Phlebomegaly	–	36 (100)^a^

*SD* standard deviation

^a^ Percentage of available data

^b^ Twelve patients (27.2 %) were on antihypertensive drugs

### Phenotype

**Fig. 1:**
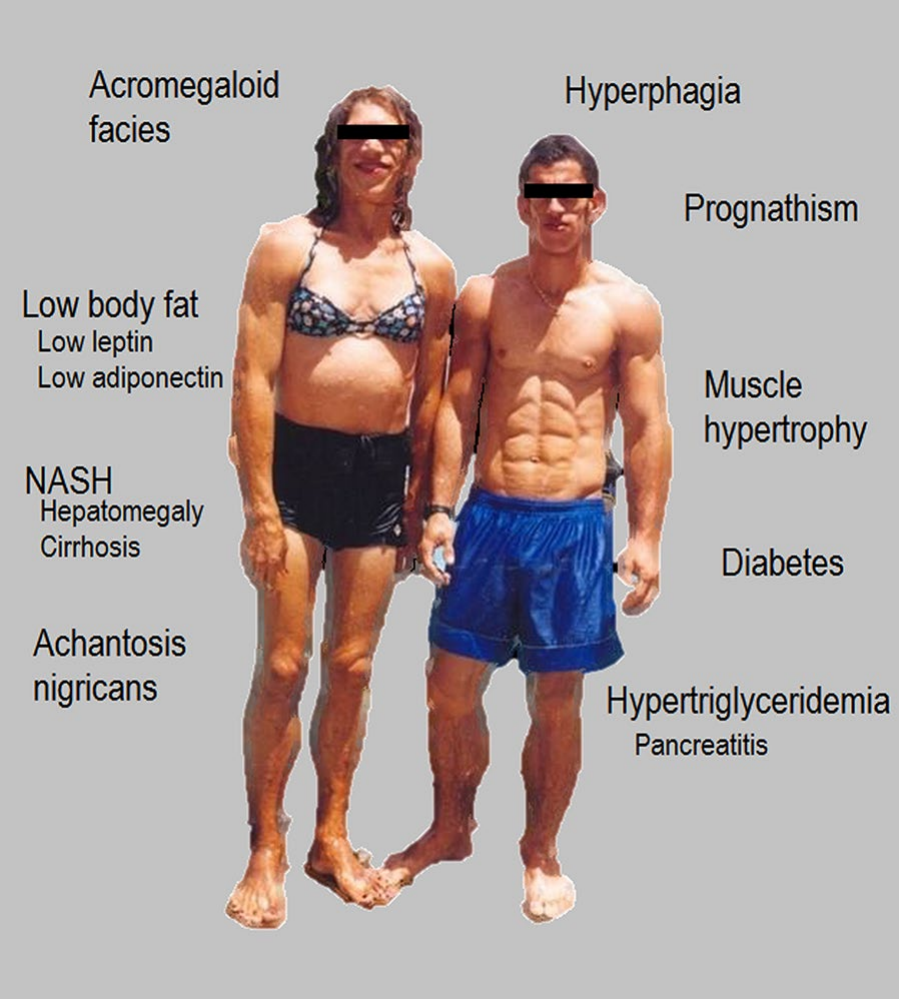
Clinical features of BSCL—Two patients with BSCL type 2. Acromegaloid facies, prognathism, atrophic cheeks, abdominal distension (female)

**Fig. 2:**
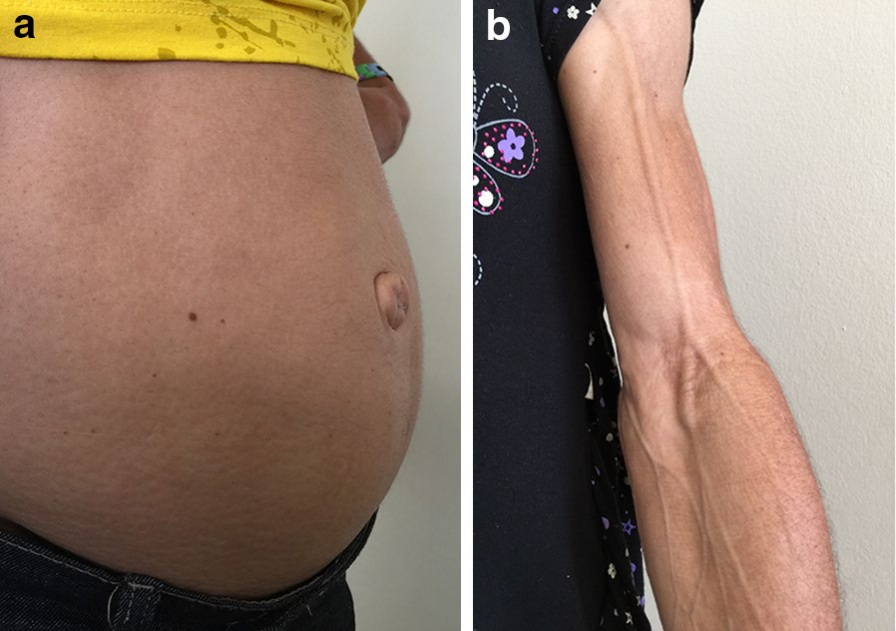
**a** Abdominal distension (hepatomegaly), umbilical protrusion, and phlebomegaly **b** in two patients with Berardinelli-Seip syndrome

Anthropometric characteristics are described in Table 1. Only two patients were overweight (BMI 25.1 and 27.2 kg/m^2^). BMI correlated positively with age (r = 0.72, p < 0.001), waist (r = 0.86, p < 0.001), % body fat (r = 0.53, p = 0.03), systolic blood pressure (r = 0.39, p = 0.02), and HbA1c (r = 0.58, p = 0.001). Twenty patients had available data of body fat measured by DEXA analysis and, as expected, all of them had very low body fat (5.4 ± 0.8 %). On physical examination (Fig. 1), acanthosis nigricans, acromegaloid facies, atrophic cheeks (loss of Bichat’s fat ball), prognathism, phlebomegaly (Fig. 2b), and muscle hypertrophy were the most common findings. Acanthosis nigricans, a cutaneous marker of insulin resistance, was very frequent, but acrochordons, another important cutaneous alteration in insulin resistance, were present only in a minority of patients. No patient had umbilical hernia. One patient had a myocardial infarction at 18 years old, and another patient had bilateral occlusion of femoral arteries causing leg ulcers and difficulties in healing.

### Laboratory investigations

#### Biochemistry

Biochemical tests are presented in Table 2. As the majority of patients were diabetic, glycemia and glycated hemoglobin were elevated. As expected, diabetics had higher glycemia (152.0 [67.0–424.0] vs. 71.0 [66.0–86.0] mg/dl, p < 0.001) and glycated hemoglobin (8.3 ± 1.9 vs. 5.4 ± 0.7 %, p < 0.001) than non-diabetics. Glycemic control was poor. Only 9 out of 30 (30.0 %) patients with diabetes had HbA1c lower than 7 %, and the majority (16 out of 30 patients, 53.3 %) had values greater than 8 %. Hypertriglyceridemia and, consequently, low HDL were common findings. No patients had HDL above 45 mg/dl and 22 patients had HDL < 30 mg/dl. Only two patients (both were children) had normal triglyceridemia. Diabetics had higher triglyceridemia than non-diabetics (330.0 [119.0–1121.0] vs. 190.5 [124.0–422.0] mg/dl, p = 0.009). Approximately two-thirds of the patients had normal values of aminotransferase (AST < 40 mg/dl—28/44 patients, 63.6 %; ALT < 40 mg/dl—27/44 patients, 61.4 %) and GGT (29/44 patients; 65.9 %). Women had higher values of GGT than men (GGT 35.0 [11.0–552.0] vs. 24.0 [14.0–114.0] mg/dl, p = 0.019). There were no gender differences regarding AST and ALT.

**Table 2:** Laboratory findings of patients with BSCL

Test	Result	Reference range
Glycemia (mg/dl)	89 (55–424)	70–99
Non diabetic	71 (55–86)	
Diabetic	152 (67–424)	
Glycated hemoglobin (%)	7.4 ± 2.1	<5.7
Non diabetic	5.4 ± 0.7	
Diabetic	8.3 ± 1.9	
Total cholesterol (mg/dl)	170 (106–316)	<200
HDL cholesterol (mg/dl)	30.3 (18–44)	>40 (>50)
LDL cholesterol (mg/dl)	98.6 ± 40.6	–
Non HDL cholesterol (mg/dl)	147.9 ± 43	–
Triglyceridemia (mg/dl)	276 (119–1121)	<150
Non diabetic	190.5 (124–422)	
Diabetic	330 (119–1121)	
Urea (mg/dl)	26 (10–74)	15–45
Creatinine (mg/dl)	0.5 (0.3–1.4)	0.6–1.2
AST (mg/dl)	31 (13–98)	<40
ALT (mg/dl)	35 (10–210)	<40
GGT (mg/dl)	31.5 (11–552)	
Women	35 (11–552)	<38
Men	24 (14–114)	<55
Insulin (mUI/ml)	25.8 (2.0–164.0)	<16
Patients <10 years	11.0 (2.0–41.0)	
Patients >10 years	27.8 (4.9–164.0)	
HOMA_IR_	7.3 (0.3–73.8)	<2.7
Leptin (ng/ml)	1.0 (0.4–2.3)	
Women	1.0 (0.4–2.3)	<14.7
Men	0.9 (0.5–1.7)	<7.4

All comparisons between non-diabetics and diabetics have p < 0.05

*AST* aspartate aminotransferase; *ALT* alanine aminotransferase; *GGT* gamma-glutamyl transpeptidase; *HOMA* homeostasis model assessment

#### Hormones (Table 2)

Forty-two patients had insulin measured. Hyperinsulinemia was present in the majority of patients (33/42 patients; 78.6 %). Patients were classified according to age (less than 10 and more than 10 years), and we observed that older patients had more hyperinsulinemia than younger patients (90.6 % [29/32] vs. 40 % [4/10], p = 0.003). The HOMA_IR_ was consistent with insulin resistance (HOMA_IR_ > 2.7) in 33 patients (78.6 %), and correlated positively with total cholesterol (r = 0.48, p = 0.007), triglycerides (r = 0.42, p = 0.02), and GGT (r = 0.45, p = 0.012). Leptin values were very low in all patients (n = 39—Table 2); the higher value was 2.3 ng/ml (normal values for women <14.7 ng/ml and for men <7.4 ng/ml) and almost half of the patients had values lower than 1.0 ng/ml. There was no difference between children’s and adults’ leptin values (0.9 [0.5–1.1] vs. 1.0 [0.4–2.3] ng/ml, p > 0.05).

#### Genetics

Twenty-nine patients (65.9 %) had a genetic diagnosis. Mutation of the gene BSCL2 (seipin—Gng3lg669insA) was present in 93.1 % of the cases (27 patients). The other two patients were sisters and showed a mutation in the gene AGPAT2 (AGPAT2 A712T). Of the 15 patients who had no genetic analysis, one was the brother of the two patients with the mutation in AGPAT2 and probably also had this mutation.

## Discussion

We report a series of patients with BSCL. This is a rare syndrome and case series reported in the literature are usually gathered from patients of several hospitals/clinics [12, 13]. Our patients are accompanied in a single setting, the endocrinology clinic of the University Hospital Onofre Lopes, in Natal/RN, Brazil. Over the past 17 years, approximately 54 patients were diagnosed and monitored in this hospital. As consanguineous relationships exist in certain regions of our state, every year we still do some new diagnosis of this syndrome. In some cases, this diagnosis is as early as the first year of life, but in others, it may be much later, with the patient already presenting chronic complications of diabetes. We present here an overview of the clinical and laboratory findings of one of the largest series of patients followed in a single medical center, by the same medical team.

Since the phenotype of BSCL is typical (acromegaloid facies, muscle hypertrophy, acanthosis nigricans, decreased body fat, mild enophthalmos, among others), the clinical diagnosis of BSCL is relatively easy but requires a clinical suspicion (Fig. 1). Quite often, parents report that since birth or early in life the child had a different appearance, however, diagnosis is usually delayed. After our first cases, the identification of other cases was easier, since the medical staff became more aware of the potential cases. In addition, families have become pro-active, further facilitating early diagnosis.

Consanguineous marriages are not rare, occurring in about 10 % of the world population [14]. This significantly increases the risk of developing genetic diseases, like BSCL. In fact, this is one of the most important risk factors in our study. More than two-thirds of our patients (72.4 %) reported consanguinity between parents and more than half (61.4 %) had, at least, one relative affected by the syndrome. The consanguinity rate can be even bigger; one patient only discovered the family relationship of her parents when she searched it until the fourth generation.

The majority of our patients were BSCL Type 2. This could help to explain the low average age of the patients (21 years) since this type is more severe and has a greater predisposition to premature death. AGPAT2 gene is essential in lipogenesis, while BSCL2 is also involved in the differentiation of pre-adipocytes and adipocyte maturation. In this setting, BSCL Type 2 has the most severely affected phenotype, with an almost complete lack of body fat [15, 16]. We can divide the adipose tissue into metabolic (subcutaneous, intermuscular, intra-abdominal, intrathoracic, bone marrow, etc.) and mechanical (palms, soles, orbits, periarticular). Both (metabolic and mechanical) are decreased in Type 2, while mechanical fat is commonly preserved in Type 1 [6, 15].

Recently, seipin has been described with functions beyond those in adipose tissue. It seems to be an important protein in spermatogenesis as well as in the nervous system [17]. Seipin knockout rats have reduced brain weight and infertility with azoospermia [17]. Some studies have linked seipin defects with motor neuron disease [18]. Seipin is expressed in the cerebellar cortex, cerebellum, and hypothalamus, and have a significant action on neurotransmission, regulating excitatory synaptic transmission [16, 19]. There is a tendency for a reduction in brain volume in patients with BSCL2 mutations, but that does not seem to be due to brain atrophy [17]. In the literature, when evaluated using specific questionnaires, intellectual impairment was more frequent in Type 2 than in Type 1 (78 vs. 10 %) [20]. We did not assess cognitive function, but it is easy to see during the anamnesis that most BSCL type 2 have this difficulty.

We only had patients with Type 1 or Type 2 BSCL. Due to the small number of Type 1 patients (AGPAT2 gene mutation), the comparison between these types was not possible. Some clinical features can help to differentiate these types. The extreme lack of body fat together with an intellectual deficit favors the clinical diagnosis of Type 2 BSCL. Acanthosis nigricans, acromegaloid facies, prognathism, atrophic cheeks (loss of Bichat’s fat ball), abdominal distension with protruding navel, phlebomegaly, and muscle hypertrophy are other common clinical findings (Fig. 1). Acrochordon, a marker of insulin resistance, is not usual in our patients, perhaps because it is more frequent in patients with higher BMI [21] and also because adipocytes (lacking in BSCL) are usually seen in the histopathological examination of this lesion. Eruptive xanthomas are not frequent.

In the literature, there are several reports of umbilical hernia in patients with BSCL [12, 22]. None of our patients required surgery for umbilical hernia repair, and on physical examination, we did not detect any cases of hernia. In contrast, the absence of subcutaneous adipose tissue made the enlargement of the umbilicus very frequent, being present in almost all patients (Fig. 2a). Hepatomegaly may also contribute to the umbilical prominence. This umbilical protrusion can be confused with a hernia and perhaps this may have happened in some cases described in the literature, as we already published [23].

Usually, in individuals without BSCL, lipids are stored during the feeding state, and this stock is mobilized during fasting. Together with other mechanisms (hypoleptinemia, insulin resistance, etc.), low body fat would explain the increased appetite commonly seen in these patients. Quite often, parents complain about the difficulty of controlling the appetite of their children.

Diagnosis confirmation can be done by searching for one of the known mutations. Of the 44 patients studied in our cohort, 29 were genotyped. Clinical features together with laboratory and imaging (DEXA) tests can help to increase the degree of clinical suspicion. Determination of body fat by DEXA scan is simple and inexpensive. Invariably and consistent with the diagnosis, all of our patients submitted to this test had very low body fat, rarely exceeding 7 %, even in those with higher BMI. Serum leptin measurement is available in many centers with an acceptable cost. Concordant with the very low body fat, serum leptin is usually very low. The combination of clinical features, low body fat measured by DEXA and very low serum leptin can make the diagnosis. Nevertheless, we still advise looking for the mutation, because of the clinical peculiarities of each type.

The majority of our patients were diabetics (67.4 %). This is expected because diabetes usually starts early in this syndrome (mean age 15 years), and the mean age of the group was 21 years. Diabetes was not detected in any affected children under the age of 10 years, and their serum insulin was lower than that of adults. Interestingly, even with higher values of serum insulin, adults develop diabetes. This may indicate that, unlike Type 2 diabetes where the failure to increase insulin secretion by the beta cells is an important factor to the onset of the disease [24, 25], in patients with BSCL, the increasing insulin resistance appears to be the key determinant. We could hypothesize that with age and difficult to deposit fat in the adipocyte, the lipids are stored in the liver and muscles, reducing insulin sensitivity and requiring higher secretion of the beta cells [22]. These cells continue to secrete too much insulin, but it does not seem to be enough, and the patient develops diabetes. Serum leptin did not differ between adults and children, being very low in both groups. This confirms that, as an autosomal recessive hereditary disease, BSCL has already started since birth, in contrast to insulin resistance that worsens with age.

The glycemic control of these patients was not good, but this is not a peculiarity of this syndrome. Worldwide and specifically in Brazil, only a small percentage of diabetic patients has good glycemic control. The analysis of a series of more than 5000 type 2 diabetic patients in Brazil showed that the mean HbA1c was 8.6 % and only 26 % had HbA1c <7 % [26]. The data of our patients were very similar (mean HbA1c 8.3 % and only 30 % with HbA1c <7 %). In recent years, we have been diagnosing the BSCL earlier (some cases even before 1 year of age). We hope this will allow imposing actions that will delay the onset of diabetes, prevent its complications, and improve life expectation.

Hypertriglyceridemia was present in the great majority of patients (42 patients, 95.5 %), being higher in diabetics (330.0 [119.0–1121.0] vs. 190.5 [124.0–422.0] mg/dl, p < 0.05). This is the result of serum accumulation of chylomicron and VLDL as well as excessive hepatic production due to liver steatosis [27]. The tissue uptake of triglycerides is defective due to the inability of adipocytes to store energy. Moreover, the lipolysis caused by endothelial lipase and stimulated by insulin is attenuated due to insulin resistance [27]. Although hypertriglyceridemia is common and acute pancreatitis occurs in some patients, this is not a usual cause of death. On the other hand, hypercholesterolemia is not so frequent, and when present, it is mild. Less than one-fourth of patients (10 of 44, 22.7 %) had total cholesterol greater than 200 mg/dl. But we know that patients with hypertriglyceridemia have a higher concentration of small and dense LDL [28] and, consequently, a higher cardiovascular risk. This can be confirmed in our patients who had a myocardial infarction and atherosclerotic leg ulcers at an early age. HDL cholesterol is always very low, as expected in patients with hypertriglyceridemia, and no patient had HDL higher than 45 mg/dl. Hypertriglyceridemia, low HDL, and small and dense LDL are essential parts of the atherogenic triad seen in metabolic syndrome and also in our patients [28, 29]. Improvements in the lipid profile seen in patients using metreleptin (an analog of human leptin) confirm the important role of leptin in the treatment of dyslipidemia [30]. The mechanisms by which leptin improves insulin resistance are not yet known [22]. It seems that there is a reduction in fat deposition in the liver and muscles [31]. None of our patients was on metreleptin. Despite its approval for BSCL in other countries, metreleptin is not yet available in Brazil.

Hepatic steatosis is part of the syndrome, but liver enzyme elevation was not frequent in our series. Two-thirds of the patients had liver enzymes within the normal range. We must remember that the aminotransferases are not reliable predictors of steatohepatitis and several patients with normal aminotransferases may have steatohepatitis when undergoing liver biopsy [32].

## Conclusions

We report one of the largest series of patients with BSCL treated at a single medical center. The lack of genetic analysis in one-third of the patients is a limitation to the best clinical characterization of the group. We believe that since the majority of patients were from the same region, two-thirds of the patients who already had genetic diagnosis must represent the proportion of the types of BSCL in this population. We hope that earlier clinical/genetic diagnosis and more efficient treatment options can reduce complications, prevent early deaths, and thus increase the expectation and quality of life of these patients.
